# A look back at the strike by Mozambican doctors in 2013: what can we learn?

**DOI:** 10.1186/s12913-024-11998-7

**Published:** 2024-11-29

**Authors:** Alexandre Lourenço Jaime Manguele, Isabel Craveiro, Mohsin Sidat, Dulnério Barbosa Sengo, António Jorge Rodrigues Cabral, Paulo Ferrinho

**Affiliations:** 1 Instituto Superior de Ciências de Saúde, Maputo, Moçambique; 2 Global Health and Tropical Medicine, GHTM, LA-REAL, Instituto de Higiene e Medicina Tropical, IHMT, Universidade NOVA de Lisboa, Lisboa, Portugal; 3 Faculdade de Medicina, Universidade Eduardo Mondlane, Maputo, Moçambique

**Keywords:** Doctors, Healthcare workers, Strike, Protests, Health services, Mozambique

## Abstract

**Background:**

The occurrence of strikes in the health sector has been an increasing concern around the world, given their negative impact on the provision of services and care to patients. The Mozambican doctors' strike in 2013 2013 is considered by many to be the largest of a kind in the country's history, and marked the changes which are still a matter of debate. The aim of this study is to understand the causes, strategies and perceived impact of this strike from the perspective of the main actors involved, taking a look back at everything that happened, including the backstage and tense moments during the negotiations. These details have been little covered in similar studies and are important for a better understanding and management of this type of movement.

**Methods:**

This is a qualitative study with a phenomelogical approach that consisted of semi-structured interviews with the main players involved in the strike movement, and analysis of documents produced around this movement. Non-probabilistic snowball sampling was used to select participants until data saturation was reached. The interviews were transcribed and imported into Nvivo version 12, and the data was analysed using content analysis to identify themes related to the research questions.

**Results:**

The doctors were demanding better salaries, career prospects and working conditions. Failure to fulfil agreements, threats from the government and a lack of communication are believed to have precipitated the strike. Faced with staff shortages, the government restricted services, prioritised urgent cases, and patients saw services slowed down, their care delayed, a lack of medicines in health units and a loss of confidence in the healthcare system. Although the strike contributed to the approval of the Doctors' Statute, it led to the interruption of postgraduate studies, transfers and suspensions of professionals.

**Conclusion:**

The strike was motivated by aspects associated with salaries and working conditions. Some of the approaches adopted further distanced the parties and delayed consensus. The strike had negative consequences for everyone, especially patients. This study provides important lessons for improving strike prevention and management strategies in the health sector.

**Supplementary Information:**

The online version contains supplementary material available at 10.1186/s12913-024-11998-7.

## Background

Strikes are mechanisms adopted by employees to force specific demands or claims when the possibilities of reaching an agreement with the employer through negotiated means have been exhausted. Strikes imply interrupting, partially or completely, the provision of services for a few hours, several days or longer [1–3].

The occurrence of strikes by doctors and other healthcare workers (HCW) has been a frequent concern around the world, given their negative impact on the provision of healthcare to patients, and the political, organizational and financial implications they bring [2, 4].

Wage arrears, non-payment of allowances, lack of opportunities for professional development and career progression, poor working conditions, shortages of essential medicines, as well as aspects related to political opposition and leadership have all been reported as triggers for HCW's strikes [1, 5–7].

**Fig. 1:**
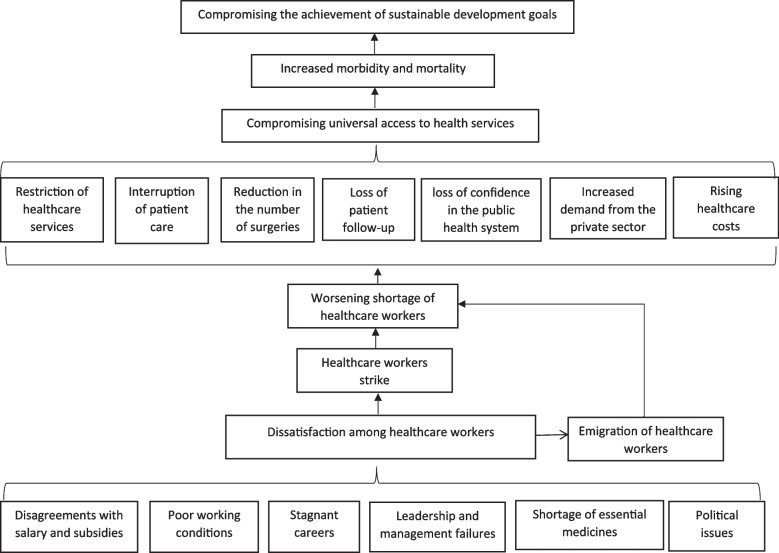
Conceptual diagram of the causes and consequences of the strike

Several implications of HCW's strikes for health services and patients have been reported in published studies, including interruption of patient care, reduction in the number of surgeries, prolonged hospitalization, loss of patient follow-up, worsening of patients' state of health, increased morbidity and mortality, search for alternative health services, high rates of referral to private hospitals, high private hospital costs, loss of confidence in the public health system and emigration of qualified HCW (as can be seen in Fig. 1) [1, 8, 9].

The effects of HCW's strikes seem to be worse in low- and middle-income countries due to already underlying limitations regarding infrastructure and resources, weak institutional arrangements and a lack of affordable alternative healthcare outlets [1, 10].

Strikes aggravate pre-existing human resources for health crisis in sub-Saharan African (SSA) countries. This compromises the achievement of universal health coverage, as well as of the Sustainable Development Goals [11, 12].

In 2013, there was a doctors' strike in Mozambique, the motivations and consequences of which have never been systematically studied, and there are no published studies on this topic in Mozambique. This strike took place in two periods, the first from the 5th to the 15th of January 2013, involving only doctors, and the second from the 20th of May to the 20th of June of the same year, when the doctors mobilized other HCW groups to support their fight.

During the strike, the doctors were represented by the Mozambique Medical Association (AMM, acronym in Portuguese), while other HCW (such as nurses, pharmacy technicians, laboratory technicians, service agents and other health technicians) were represented by the United Health Professionals (PSU, acronym in Portuguese), both currently registered as trade unions.

At the beginning of the strike, the PSU, unlike the AMM, was an unofficial association, that is, all legal procedures for its creation had not been followed [13]. The PSU was created in response to the non-adherence to the strike by the National Association of Nurses of Mozambique (ANEMO, acronym in Portuguese) and the Association of Laboratory Technicians and Clinical Analysis (ATLAC, acronym in Portuguese) [14], and ANEMO was accused of acting clandestinely and passively, neglecting the defence of the class [13].

Therefore, this study aims to understand the process of the 2013 doctors' strikes in Mozambique, including the contextual factors that contributed to the strikes, motivations and demands of HCW, tactics used by public health authorities and the government to mitigate its effects and/or stop it, and its consequences for the public, HCW and the public health system. In addition to documenting the findings and to contributing for the analysis of the topic in a scholarly manner, the study also aims to be an evidence base for health sector policy and decision makers to guide strategies and policies to manage and control strikes, as well as anticipate and prevent their occurrence in Mozambique and other countries with similar socio-economic conditions.

## Methods

### Study location

Mozambique is a country in south-east Africa, bathed by the Indian Ocean to the east and bordered by Tanzania to the north; Malawi and Zambia to the north-west; Zimbabwe to the west and the Kingdom of Eswatini and South Africa to the south-west. In its government structure, the country has several ministries, including the Ministry of Health (MISAU, acronym in Portuguese), which is responsible for guaranteeing access to health services and care for the entire population. The governance of the health system is being progressively decentralized to provinces, municipalities and district health authorities [15].

In 2013, Mozambique had a total of 1,452 doctors (one per 17,235 inhabitants) and 6,395 nurses (one per 3,913 inhabitants). This number increased to 2,745 doctors (one per 11,232 inhabitants) and 10,268 nurses ( one per 3,003 inhabitants), in 2021 [16, 17].

### Study design

This is a qualitative study with a phenomelogical approach that consisted of semi-structured interviews with the key actors involved in the Mozambican doctors' strike movement in 2013 (government leaders, civil society and HCW organizations) and analysis of official and media documents issued in the context of the doctors and HCW’s strikes [18–21].

### Data collection procedures and tools

#### Semi-structured interviews

Data were collected between March 2022 and May 2023. Semi-structured in-depth interviews were carried out based on an interview guide prepared, pre-tested and revised with the help of a panel of experts with extensive experience in qualitative research. The pre-test helped to ensure better adequacy and focus of the questions to the research objectives, as some questions were a little vague, so they were reformulated. Some questions were very similar, with a certain redundancy, so they were eliminated or combined.

The interview guide comprised open questions to better understand the determinants and contours of the Mozambican doctors' strike in 2013 and included questions about: 1) the purpose of the strike; 2) the main actors involved; 3) the implications; 4) the tactics adopted; 5) the repercussions and the resolution of the strike (as can be seen in the supplementary material 1). During the interviews, the interviewers were allowed to add questions, while the interviewee was allowed to delve deeper into issues that they considered pertinent to a better understanding of the strike movement. The interviews lasted an average of 45 min.

Initially, the most prominent actors in the media during the strike were intentionally selected (the leaders of the strikers' group, government representatives and civil society actors), who made up an initial sample of eight (08) interviewees (including the first author A.L.JM., as minister of health on the date of the facts).

This study began with the application of an episodic interview exclusively to the first author (A.L.J.M., as minister of health at the time of the strike) to minimize a potential risk of bias, since the first author also lived through the episode to be investigated, as a preponderant figure, so the use of his information is complex and requires a different methodology [22]. Therefore, the episode interview was carried out separately in October 2021 (by I.C.), to gather information from the former health minister before the interviews with the other participants began, and even before the process of re-reading and analyzing the documents written by him at the time of the strike, in order to minimize influences on the way he experienced the phenomenon under study, as described by Flick [22].

The other interviews were conducted by a trained researcher (Celso Soares Give, trained by I.C.). Celso Soares Give is a senior lecturer and researcher affiliated to the community health department of the Eduardo Mondlane University in Mozambique, with a Master's degree in Public Health, who has dedicated himself to carrying out qualitative research, so he is familiar with the application of interviews in this field, while I.C. (who conducted only the episode interview) is a sociologist, teacher and researcher with mastery and experience of interview techniques in different contexts. None of the interviewers was involved in the strike movement in any capacity.

It was important that the interviews (with the exception of the episodic interview) were carried out by a researcher external to the study, as a strategy to control any kind of influence (personal, interpersonal or political) that the study might suffer (described in the research team's reflexivity in supplementary material 2).

**Fig. 2:**
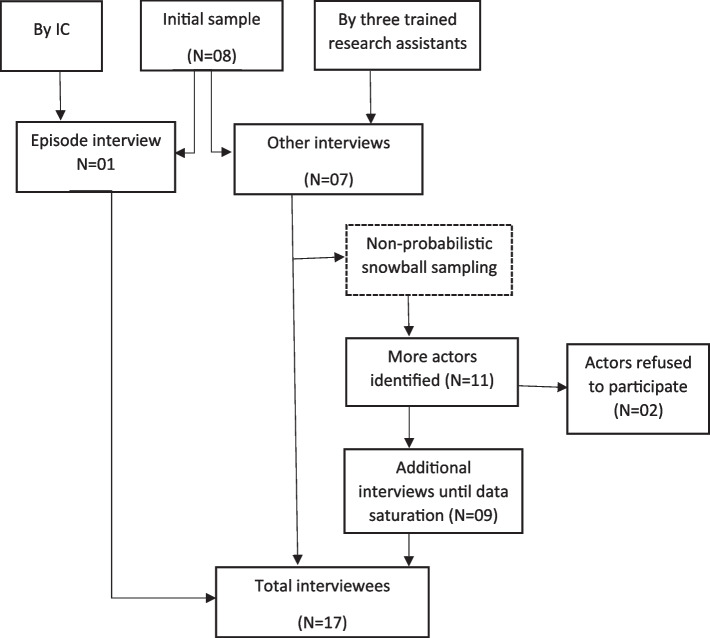
Summary of the interview process

During these interviews, more actors (*n* = 11) were identified through a non-probabilistic snowball sampling conducted until data saturation. No new data emerged from the 13th interview onwards, and the subsequent three interviews confirmed the achievement of data saturation [23–26]. Of a total of 19 actors identified, only two actors, one from the striking group and the other from the government, refused (without justification) to participate in the study, giving a refusal rate of 10.5%. Therefore, a total of 17 interviews were carried out, including the episodic interview (as can be seen in Fig. 2).

All interviews were conducted in Portuguese and recorded with the prior written consent of the interviewees. The interviews were conducted mostly in person, but in four cases virtually (via Zoom), depending on interviewees’ availability.

#### Document analysis

**Fig. 3:**
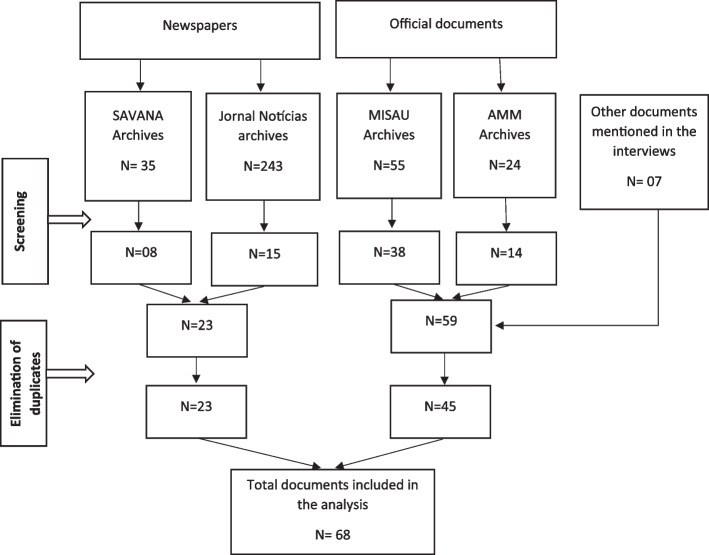
Flowchart of the document selection process

The archives of the MISAU, the AMM and two prominent printed media in Mozambique were manually searched (by Arménia Leia Sofar Mucavele, Dalmázia Helena Mariza de Castanheira e Cossa, Maria Luísa Miguel Falcão and Rosa Langa) to identify relevant documents and publications from December 2012 to July 2013. A total of 357 documents were identified and submitted to the selection process. Documents whose title or subtitle addressed the strike in the health sector were selected. To these were added documents cited by the interviewees or related to their comments (as can be seen in Fig. 3).

Therefore, a total of 68 documents were selected and analyzed, which included: media publications (two newspapers), Annual Health Human Resources Report, Pre Notice and Strike Guidelines for doctors and HCW and the respective demand books, General Report on the paralysis of activities in the National Health Service, Memorandum of Understanding (MoU) between the MISAU and AMM, Regulation and Statute of Doctors in Public Administration, AMM Regulation, Dispatches from the Minister of Health, book of minutes of meetings between MISAU and AMM, book of minutes of meetings of the MISAU Management Collective, Report of Disciplinary Processes raised in the context of the work stoppage, Constitution of the Republic of Mozambique of 2004, Regulation of the General Statute of State Employees and Agents, and Statute of the Mozambican Doctors Council (OrMM, acronym in Portuguese) and Code of Ethics (as can be seen in the supplementary material 3: Supplementary Table 1).

### Data analysis

The interviews were transcribed verbatim by a researcher external to the study (Sofia Arminda Roberto) and then checked (by I.C.). In the end, the preliminary transcripts were shared with the interviewees for checking, and after four weeks, the interviewees were telephoned for additional comments (with the exception of the episode interview), which were recorded, transcribed and included for analysis [27]. All interviewees were involved in this process, but only three interviewees (18.8%) made additional comments.

The final (anonymised) transcripts were shared with the researchers (A.L.J.M., D.B.S. and M.S.) so that they could proceed with the analysis, first independently and then together to ensure better alignment between them [28]. All analyses were carried out using the Nvivo programme (version 12).

The content analysis technique was used to analyse the documents and the interviews’ transcriptions, identifying the emerging core in each text, through the process of coding (categorisation) and interpretation of the information contained, to uncover its manifest and latent content, according to the procedure described by Bengtsson [29]. Initially, through a quick review of the literature, a set of themes and subthemes were predefined, and others were added during the analysis of the transcripts and documents (Table 1).

**Table 1:** Summary of themes defined during analysis

Themes	Subthemes
	Income and living conditions
Causes	Working conditions
	Socio-political conditions
	Government’s approaches
Approaches adopted during the strike	Strikers' approaches
	Mediators' approaches
Impact of the strike	For the National Health System, services and for users
	For doctors and other healthcare workers
	Changes achieved with the strike

The researchers then read and re-read the transcripts as a whole to familiarise themselves with the data. The transcripts were broken down into smaller meaning units. These units are constellations of sentences or paragraphs containing aspects related to the causes, tactics adopted and impact of the strike. Each meaning unit was labelled with a code according to the pre-defined list (deductive) and others that emerged from the data (inductive). This process was repeated twice, starting from a different page each time to increase stability and reliability. Subsequently, the transcripts were read again together with the final list of meaning units and each meaning unit was marked on the original transcript. The unmarked text was re-evaluated to see if any important aspects had been missed. Then, the number of meaning units was reduced by condensing them. The meaning units were grouped into causes, tactics (strategy) and the impact of the strike, taking into account the research questions, so the meaning units were transformed into sub-themes, and their groupings into themes. After this phase, the researchers (A.L.J.M., D.B.S. and M.S) met to analyse the categorisation made by each of them and eliminate discrepancies. The data was compiled and analysed in manifest and latent form, and in each theme and sub-theme, meaning units were chosen that best represented the manifest or latent content and presented in quotations [29].

The recording of the episode interview (with A.L.J.M.) was shared at the end [22], after the analysis of the other interviews had finished, so it was transcribed verbatim (by Sofia Arminda Roberto) and analysed (by A.L.J.M., D.B.S. and M.S.), serving as a complement for a better understanding of the facts.

### Ethical considerations

This study was approved by the Institutional Bioethics Committee of the Instituto Superior de Ciências de Saúde (on 21 August 2020, ref. 02/CIBS-ISCISA/2020) and by the Ethics Committee of the Instituto de Higiene e Medicina Tropical, Universidade Nova de Lisboa (on 06 April 2021, ref. 2.21). All the interviewees were previously informed about the nature of the study and participated by signing an informed consent form. They were also informed that their participation was voluntary and that they were free to withdraw their consent to participate in the study at any time, without conditions or reprisals. The anonymity of the participants was guaranteed by coding their names (E1, E2… E17) in the interviews, transcripts and manuscript. The interviews took place in a private environment of each participant's choice and only in the presence of the interviewer and the interviewee. Twelve months after the end of the study, the interview recordings and informed consent forms will be destroyed by the principal investigator. The study data, which will be protected by the principal investigator in electronic files, may not be used for any other type of study or for any purpose other than that inherent to this study. In these terms, this study followed the principles of the Declaration of Helsinki [30].

## Results

The interviewees were all based in the capital city, Maputo, aged between 36 and 93, with an average of 58.6 years (SD:12.8), and the predominant gender was male (70.6%). The majority were surgeons (23.5%), specialist public health doctor (23.5%) and general practitioners (17.6%), as shown in Table 2.

**Table 2:** Socio-demographic characteristics of the interviewees

Variables	N	%
**Age** (average: 58.6 years)		
31–40	2	11.8
41–50	2	11.8
51–60	6	35.3
> 60	7	41.2
**Gender**		
Male	12	70.6
Female	5	29.4
**Group belonging during the strike**		
Striking healthcare workers	6	35.3
Mediators (civil society)	5	29.4
Employers (Government)	6	35.3
**Occupation**		
Surgeon	4	23.5
General practitioner	3	17.6
Internist doctor	1	5.9
Specialist public health doctor	3	23.5
Nurse	1	5.9
Religious leaders	2	11.8
Manager	1	5.9
Lawyer	1	5.9

### Antecedents of the doctors’ and HCW’s strike in Mozambique

On 7 December 2012, the AMM sent a nationwide strike notice to the MISAU, which would begin on 17 December 2012 [31], since then a cascade of key events have taken place (Table 3).

**Table 3:** Summary of the main events

Chronology of events		Date
1	Approval of the Doctor Statute by the 22nd Ordinary Session of the Council of Ministers	07/21/2012
2	Pre-strike notice by AMM in representation of doctors	12/07/2012
3	Talks begin between MISAU and AMM	12/07/2012
4	Postponement of strike after reaching some points of consensus	12/14/2012
5	Creation of the Statute Revision Committee and the Salary Technical Committee	12/17/2012
6	Mozambique doctors' strike begins	01/05/2013
7	Negotiations resume	01/09/2013
8	MoU signed between the MISAU and AMM	01/15/2013
9	Mozambique doctors' strike ends	01/15/2013
10	Approval of the revised Doctor Statute by the 4th Ordinary Session of the Council of Ministers	02/26/2013
11	Pre-notice of strike for doctors and other HCW	05/13/2013
12	Start of talks between MISAU and AMM and PSU	05/16/2013
13	Start of strike by doctors and other HCW	05/20/2013
14	Suspension of postgraduate studies (specialization)	06/05/2013
15	Doctors’ and other HCW’s strike ends	06/20/2013
16	Approval by the Assembly of the Republic of the Medical Statute	11/01/2013
17	Approval of the Regulations for the Statute of Doctors in Public Administration	08/29/2014

*AMM* Mozambique Medical Association, *HCW* Healthcare workers, *MISAU* Ministry of Health, *PSU* United Health Professionals

The strike consisted of doctors paralyzing their activities in all sectors, except for emergency services at central and provincial hospitals, pediatric, gynecological and obstetric emergency services, emergency operating theatres and emergency services at health centers. The doctors were demanding better salaries and a revision of the Statute of Doctors in Public Administration (approved by the Council of Ministers on 7/21/2012, without either the AMM's or the OrMM’s opinion). They wanted some rights safeguarded in the Statute [31, 32].

Following the strike notice, talks began immediately between the Mozambican government (represented by MISAU) and the AMM, and two independent working committees were formed: The Statute Review Committee and the Technical Salaries Committee [33]. While these negotiations were ongoing the starting date of the strike was suspended. Nevertheless, the lack of consensus on salary issues triggered the beginning of the strike on 5 January 2013. This only ended with the signing of a MoU between the parties [33].

The MoU agreed to renounce threats, intimidation and future reprisals against doctors and trainee doctors as a result of the work stoppage, and the approval (by Decree and Ministerial Diplomas) of the Statute of Doctors in Public Administration and the respective revised salary scale by April 2013 [34]. The Doctors' Statute guaranteed doctors the right to housing, allowances (risk and seniority) and a different career regime within the civil service [35, 36].

On 20 May 2013, the doctors, this time accompanied by other HCW, represented by the AMM and PSU, respectively, began a national strike (under the slogan: caring for those who care), motivated by the government's failure to comply with the terms of the MoU signed in January 2013, namely: no reprisals against doctors and trainees, approval of the Doctor's Statute in the first session of the Assembly of the Republic and permanent dialogue [13, 37].

According to the strike guidelines announced by the AMM [37], the strike consisted of paralyzing the activities of doctors in all sectors, i.e. all doctors from general practitioners, academics, researchers, dentists, specialists and those with non-clinical activities in public institutions, such as the MISAU or other sectors, with minimum services being the responsibility of military doctors, foreign doctors and doctors in management positions. In districts where there were no foreign doctors, it would be up to the government to allocate the available foreign and military doctors.

According to the guidelines announced by the PSU, the strike consisted of paralysing the activities of HCW in all sectors, that is, all service agents, security guards, drivers, nurses, technicians, administrators, lecturers and others; they should not attend their workplace. The minimum services were ensured by foreign HCW, military personnel and those in management positions [13].

### The different actors involved in the strike movement

#### Strikers

The first strike (from 5th to the 15th of January 2013) was exclusively for doctors and, according to the interviewees, the non-involvement of other HCW was a preference of the doctors themselves. This changed in the second strike (from the 20th of May to the 20th of June 2013), which involved other classes of HCW.

E01 (Doctor, government representative): *''The first strike was by doctors and when they saw that it wasn't having the desired effect, they tried to mobilize nurses and other classes to get as many people on strike as possible and they called it a strike by health professionals''.*

The leaders of the strike movement were young doctors with some professional experience who had already been exposed to the reality of medical practice in Mozambique, and some were even in the process of specialization.

E10 (Doctor, government representative): *''The forces that appeared most engaged were young people, but not recent graduates, they were young people with some experience, they had already experienced some of these difficulties on a day-to-day basis. It's interesting that many of these young people were doctors in postgraduate training (specialization). There were also some older adults, eh!, I wouldn't say in leadership, but in encouragement to strike''.*

But among the strikers, not all worked in the public health sector, some worked in Non-Governmental Organizations (NGOs).

E12 (doctor, strike leader): *''I and the president of the AMM put our interests aside to the benefit of the collective, although many thought it was for financial reasons, but no, the president (of the AMM) for example, was in an NGO, to say that in 2013 he earned [a significant amount in USD], that amount is still something today, so he was well paid''.*

E17 (episode interview, government representative): *"We heard that many of the strikers were NGO workers, they even took vacations from work to come here to guide the strike, this was the case with their leader”.*

The first strike was also attended by trainee doctors (in their final year of medical school), as they could be effective members of the AMM [38].

E11 (Doctor, government representative): *"They [trainee doctors] were being pressurized to go on strike, I heard that they were even being threatened if they attend the internship''.*

#### Employers (the Mozambican government)

On the government side was the MISAU, represented by the Minister of Health, the Permanent Secretary, the Inspector General of Health and the respective National Directors, the Director General of Maputo Central Hospital, representatives of the Ministry of Public Service, the Ministry of Finance and the Ministry of Justice [33].

E17 (episode interview, government representative): *"*A priori*, the government thought that it was the responsibility of the Ministry of Health to resolve this issue, but then realized, due to the course of events, that some issues addressed were not within the responsibility of MISAU, therefore, they should be assessed by other sectors, therefore, it required greater intervention and involvement from other governmental sectors".*

#### Mediators

The mediators' mission was to bring the two parties (government and HCW) together. Some mediators acted directly, even accompanying the negotiating teams, such as the Mozambican Doctors' Council (OrMM), the Mozambican Workers' Organization—Central Trade Union (OTM-CS) and the Human Rights League [39], and later, as part of the government's tactic to sensitize the strikers, religious leaders were included. Some civil society organizations such as the League of Non-Governmental Organizations in Mozambique (Joint, acronym in Portuguese), ANEMO, ATLAC and the Mozambican Lawyers Council intervened in the negotiations in order to bring the parties together and resolve impasses in negotiations.

E01 (doctor, government representative): *"The government negotiated in good faith and there came a time when we saw that we weren't getting the results we wanted, so we included religious leaders, civil society, and other people who could help in the negotiation."*

E15 (doctor, OrMM, mediator): *“The OrMM was there (at the negotiating table) but as an attempt to bring the parties together, these parties, in this specific case, were the Government and on the other side were the doctors.”*

E07 (doctor, striking group): *‘’The president of the Human Rights League supported us a lot, yeah!, then some entities appeared, some personalities tried to talk to us to try to get us to stop the strike and continue the dialogue: that is the case of the President of the Lawyers' Council’’.*

E17 (episode interview, government representative): *‘’When I realized that the Human Rights League was on the strikers' side, on the day I had to meet with the striking doctors I also asked for the Human Rights League to be present’’.*

### Causes of the doctors’ and other HCW’ strike

From the strikers’ perspective there were three types of causes underlying the strike: those associated with living conditions, working conditions and socio-political conditions (Table 4).

**Table 4:** Summary of the main causes of the strike

**Themes**	**Subthemes**	
**Causes**	Income and living conditions	• Lack of right to housing
		• Salary gap (compared to other sectors)
		• Low salary
		• Non-payment of allowances; • No career progression
	Working conditions	• Insufficient biosafety equipment
		• High workload
		• Lack of medicines and supplies
		• Inadequate infrastructure
	Socio-political conditions	• Regulation of the Doctor's Statute
		• Lack of communication with healthcare workers
		• Lack of transparency in public fund management
		• Lack of investment in the healthcare sector
		• Lack of trust in government
		• Negative influences of foreign policy stakeholders

#### Income and living conditions

Aspects associated with the living conditions of doctors and other HCW were cited as the main triggers for the strike by comparison with the salaries of other professional classes such as judges, prosecutors and fiscal technicians, so doctors and other HCW were demanding better salaries, including risk and seniority allowances, as well as the right to housing (especially for HCW who worked far from their usual place of residence).

E07 (doctor, strike group): *"We demanded a salary similar to that of magistrates and a risk allowance different from the one stipulated at the time, which I think was 7.5 or 10 per cent of the monthly salary; so we wanted it to be between 25 and 30 per cent, taking into account the risks, the exposure we are subject to; another thing was to be given the right to have a furnished house with expenses paid for by the state, with water, electricity bills all paid for by the state; and the approval of our statute."*

Another aspect cited by the interviewees that motivated the strike was the lack of progression in their professional careers.

E14 (nurse, strike group): *‘’The problem of career progression was a major factor for us, we made little progress, so it made the nurses angry too and they supported the strike’’.*

#### Working conditions

HCW complained of a lack of infrastructure, medicines, diagnostic and protective equipment to carry out their activities, as well as a high workload.

E04 (doctor, strike group):* "I'm talking about the day-to-day of being a doctor, arriving at a Health Unit and seeing patients without the right conditions for the patient and for us too, from infrastructure to working resources and diagnostics".*

E12 (doctor, strike group): *"We had a heavy workload, in other words, for example, I was in a sector where I did 24-h emergencies, then went to the operating theatre, and after operating, I had to see the patients on the ward and then go home, so that the next day I could be there at 8am to work".*

#### Socio-political conditions

The first strike ended with the signing of a MoU between the AMM and the MISAU (on 15 January 2013), with some terms and deadlines for compliance, but some of these terms were not met by the agreed deadlines, and the government's openness to dialogue decreased, showing a certain insensitivity towards the HCW' problem, which led to the second strike.

Doctors wanted to be treated with distinction within the civil service, as a way of valuing the profession and giving it prestige in society, and they had the doctors' statute as a guarantee, which is why they demanded that it become law (being approved by the Assembly of the Republic) and within the established deadlines [34, 36].

In the strike guidelines (dated 14 May 2013), the AMM refers to three unfulfilled points: 1) No reprisals against doctors and trainee doctors; 2) Approval of the Doctors' Statute in the first session of the Republic Assembly; and 3) Permanent dialogue and joint action to achieve the established goals [37].

According to the strikers, dialogue between the parties had become even less fluid since the initial strike. Despite continuous insistence on the need for dialogue on the part of AMM, silence was always the response from the competent entities (government) [37].

E07 (doctor, striking group): *“There was a violation of the MoU by the government and the deadlines were not met, which is why we went on a second strike.”*

Another aspect cited by the interviewees was the lack of transparency in the management of public funds, with no clarity in the criteria for allocating funds, clearly disadvantaging the health sector.

E04 (doctor, striking group):* "We saw that the application of funds wasn't judicious and transparent, with an imbalance that affected the health area and didn't allow it to develop, so we saw that health wasn't a priority".*

However, it was also believed that there was external influence, that the strike movement was inspired by similar movements abroad.

*E17* (episode interview, government representative): *''This movement had a greater dimension because there were already a good number of Mozambican general practitioners and specialists, and it was organised in detail. I think it was organised based on experiences of what we see happening in other countries''.*

Among members of the government, there were those who believed that the strikers had external support and that the movement was linked to the fulfilment of a foreign and domestic political agenda, precisely because of the timing of the strikes (in the run-up to the 2014 general elections), which was denied by the strikers.

E01 (doctor, government representative): *'' The strike was more of a political movement because there were clearly signs of subversion on the part of some countries, ambassadors and NGOs that openly supported the strike's mentors, and used embassies for meetings and there was political exploitation of others. It was expected that services would be paralyzed at a national level and this would provoke disproportionate discontent among the population against the government and this would create a fury among the population with very negative consequences, such as deaths and more''.*

E05 (doctor, government representative): *‘’If we increased salaries in the health sector, then there would come a strike by the teachers and police, who were already organized, that was the scenario and we were a year away from the elections, the elections were for 2014, do you realize the implications?’’.*

*E12 (doctor, leader of the strikers): "There was miscommunication and the basis of the miscommunication was the misinterpretation of the causes of the strike as political or personal. There were never any political aspects, there were always social aspects, the issue of doctors' and HCW' salaries and the working conditions that were deteriorating, these were the two reasons, I repeat there were never any political reasons, so much so that we had a meeting with leaders of the ruling party to explain that 'look, nobody here has anything against the party, what we want is salary and working conditions and we're not against the leadership, what happens is that now the pot has burst, that's all’’.*

One aspect cited by the strikers as a catalyst for the strike was the feeling of insensitivity on the part of the team representing the government towards their concerns during the negotiations.

E04 (doctor, striking group):* "The treatment we received there, from the leaders, was something that really impacted me, they said 'we were a bunch of kids', so they didn't look at us as serious people, and many of the things that were said in those meetings were too insensitive, and we wondered how people who head the Ministry of Health would say things like that to us, wow!’’.*

### Approaches adopted during the strike

During the strike, the main mechanism adopted to reach consensus was dialogue (negotiations) between government representatives and HCW, however, this was not always possible, and additionally each group adopted different strategies to achieve their objectives (Table 5).

**Table 5:** Approaches adopted during the strike by doctors and other HCW

Approaches adopted during the strike	Government’s approaches	• Ask former health ministers for advice
		• Presentation of a Doctor's Statute with compensation allowances • The use of police force
		• Readjusting the tasks of available staff • Request support from the Mozambican Red Cross and doctors from the defense forces • Restricting services and prioritizing the most critical sectors • Interrupting pre-scheduled appointments and surgeries
		• Raising awareness among students and guardians by invoking the pedagogical regulations
		• Threat of disciplinary proceedings
		• Inclusion of religious leaders and civil society organizations in the negotiations
	strikers’ approaches	• Involvement of other entities (such as embassies) • Pressurizing final-year medical students (trainee doctors)
		• Marches and demonstrations in the streets
		• Control/spy on the Ministry and Hospitals (entrances and exits)
		• Prevent other HCW from working
		• Advocate the right to strike and invoke the Constitution (legislation)
		• Use the media to pressure the government
	Mediators' approaches	• Educating and sensitizing based on ethics and religion • Listening to the parties, promoting rapprochement and dialogue • Recruit believers and people from the community to help with activities in the health units

*HCW* Healthcare workers

At the beginning of the second strike (from the 20th of May to the 20th of June 2013), there was resistance on the part of the government to include other HCW at the negotiating table with the doctors, allegedly because their association was not regulated, contrary to the wishes of the AMM, which in turn refused to negotiate.

According to the general report on the paralysation of activities in the health sector: *“The PSU, however representative it may be, as far as we know, is an entity without legal recognition in the Mozambican legal landscape, which makes it illegitimate to celebrate any agreement on behalf of all HCW in Mozambique, some of whom are already organized in associations, such as nurses, pharmacists and laboratory professionals”* [33].

Savana newspaper (edition 06–28–2013): *“At the first meeting, scheduled for May 16, the AMM Management was accompanied by other HCW who were not part of this Association, with whom the dialogue would have to take place in another moment, but AMM imposed that the dialogue could only restart in the presence of these professionals. At this meeting, when the government expressed its position of talking only with AMM, within the scope of the MoU signed in January 2013, the AMM left the meeting room and never returned, and on the 20th the second work stoppage of 2013 began''* [40].

However, the parties continued the dialogue on May 28. The government retreated, showing openness to dialogue also with the PSU, but as mere workers of the State Apparatus and not representatives of other HCW [33].

#### Tactics adopted by the government

Faced with the doctors' wage demands, the government claimed that there were insufficient funds and that the budget plan for that year had already been approved, and that its fulfilment even depended on external aid, a somewhat paradoxical argument in the eyes of the strikers.

The President of the Republic says that doctors are pressurising the government to give them money that the state doesn't have*: "HCW chose to strike to force the government to give them more money, at a time when the state budget still depends on contributions from external partners, in the order of 30 per cent. The pay issue is a general problem and not just for the medical profession"* (SAVANA newspaper, 05–31–2013 edition) [41].

For the AMM press office, a poor country doesn't import luxury cars for its leaders, it doesn't provide luxury pensions or high-level perks for its leaders. For the doctors, it's a paradox to say that the country is poor while it spends rivers of money on useless perks (SAVANA newspaper, 05–31–2013 edition) [41].

The government planned a gradual increase in the salaries of HCW in order to reach the salary required by them within a few years, which was not understood by the strikers.

General report on the paralysation of activities in the health sector: *"In April 2013, the Council of Ministers approved the general guidelines for salary readjustment in the public service for 2013. This document differentiates the readjustment in the health sector, and aims to bring the salary scale for doctors closer to that of the judiciary in a phased process. The increase approved for this year [2013] will be 15 per cent on the basic salary for doctors and 9 per cent for other HCW''* [33].

According to the guidelines for the second doctors' strike: *"The 15 per cent salary increase, which translates into an increase in the doctor's basic salary of around two thousand meticais, demonstrates the appreciation that the Mozambican government has for us—a total contempt''* [37].

E06 (religious leader, mediator): ‘’*We really all agreed that conditions should be improved, but there were no material conditions to realise this desire. So, the point of contention starts here, because the doctors, especially the younger ones, wanted to solve the problem immediately, but the country doesn't solve problems immediately, but with medium and long-term programmes’’.*

In an attempt to stop the strike, the government presented a proposal for a Doctor's Statute with some compensation allowances included, as a discreet way of increasing doctors' salaries.

There was a certain fear on the part of the government that the increase in doctors' salaries would have a domino effect and provoke strikes by other classes such as police officers, teachers and other public sector employees.

E17 (episode interview, government representative): *"I showed them the Doctor's Statute because the Ministry had no way of increasing the salary itself, so the Doctor's Statute was created as an alternative, which was a big step forward for their lives."*

*Savana newspaper (05–17–2013 edition): "On 19 December 2012, another meeting was held to discuss the issue of salaries where the AMM expressed its desire to see an increase in salary from the approximately 20,000 meticais that the younger doctors earned to 50,000 according to the table of private clinics and NGOs, with the MISAU informing that there were no conditions for the increase desired by the medical profession, the best way out being to increase the subsidies'' *[42]*.*

E01 (doctor, government representative): *"We thought there might emerge an Arab Spring and a general strike. So, when negotiating, the government also thought about the possible broader consequences of giving in".*

On the other hand, the MISAU met with Mozambique's former health ministers to seek advice on how best to manage this conflict, given their experience in governance.

E17 (episode interview, government representative): *‘’Well, I had the idea of calling former government officials to talk to them about their experience of these issues and ask for recommendations, advice… which helped a lot because they advised us on how we should approach this issue, in a cordial way and knowing that they are strikers, yes, but we are responsible for them, we exist because of them, and then understanding their cause and engaging in dialogue whenever possible, reducing confrontational attitudes as much as possible’’.*

On the ground, however, in the face of difficulties, the tactics adopted by the government were not always peaceful. At some point, the government resorted to intimidating mechanisms to achieve its objectives.

Through the Faculty of Medicine, the government pressurized trainee doctors (final year medical students) to return to their internships in hospitals by invoking article 37 of the Pedagogical Regulations of the Eduardo Mondlane University, which stipulates that the requirement for admission to a certain subject is not to have missed the equivalent of 20 per cent or more of the workload of the respective subject [43].

*E11 (doctor, government representative): ''We decided to give the students an ultimatum and told them that if they came back by that day, we would ignore everything, there would be no penalization and they would resume their internships as if nothing had happened, ok, otherwise we would penalize them by failing their internship. Some of the trainees' parents came to see us and we explained that the measure was even the mildest, we shared a copy of the pedagogical regulations, and explained that if we were strict all these kids would be expelled, but that's not what we want, we want it to be educational, but also within the rules, and the mildest measure is to fail a person who has 20% absences in a subject, repeats the subject''.*

E17 (episode interview, government representative): *''The students felt pressurized because they wanted to finish the course, and failing the internship meant a delay in their lives’’.*

Nevertheless, after the first strike ended (on 15 January 2013), the Faculty of Medicine at Eduardo Mondlane University failed the (final year) medical students who participated in the strike, ignoring the terms of the MoU signed between the AMM and the MISAU, which provoked a revolt from the AMM, which saw the Faculty of Medicine as an extension of the MISAU.

SAVANA newspaper (edition 02–01–2013): *‘’On January 30, 2013, the medical faculty of the Eduardo Mondlane University informed, in a statement, the failure of all 6th year medical students who joined the strike called by the AMM, and also determined to award certificates of recognition for selflessness and sacrifice in their academic obligations to students who did not join the strike, establishing an armistice between AMM, MISAU and the Faculty of Medicine, at Eduardo Mondlane University’’* [44].

SAVANA (edition 02–08–2013): “*Under the Pedagogical Regulations of the Eduardo Mondlane University, 6th year students, for the 10 weeks (7 days* × *10 weeks* = *70 days) of internship in the health units of the National Health Service, and due to the absence of 9 days at no time do they exceed the 25% stipulated in these regulations, which by arithmetic calculations are (9 days/70 days) 12.9% of the time after the end of the strike, the trainee doctors made up to 2 weeks' compensation for the 9 days of work stoppage*" [45].

SAVANA newspaper (02–08–2013): *‘’The spokesperson for the Ministry of Health says that her institution has no powers to intervene in this imbroglio, given that it involves the AMM and the Faculty of Medicine. she recalled that the MISAU is doing everything it can to comply with the MoU signed on 15 January between the MISAU and AMM, to avoid persecution and reprisals against doctors, but with regard to trainee doctors it can do nothing’’* [45].

Consequently, in the second doctors' strike (from May to June 2013), 6th year medical students did not join the strike, they were even assigned to provide minimum services together with foreign doctors [33].

During the strike, the available healthcare workforce was very limited, which led the government to restrict services (prioritizing the most critical sectors), interrupt pre-scheduled consultations and surgeries, readjust the tasks of the available staff, and request support from the Mozambican Red Cross and defence forces (military doctors).

Savana newspaper (07.06.2013 edition): *"Given this crisis situation, Mavalane General Hospital has been forced to interrupt treatment in some wards in order to prioritize the most critical sectors. At Maputo Central Hospital, surgeries are only being carried out on emergency patients. All pre-scheduled surgeries and consultations have been canceled" *[14]*.*

E02 (doctor, government representative):* “We had to recruit military doctors and doctors with management positions to work in Central Hospitals”.*

E017 (episode interview, government representative): *“We took the students from ISCISA (acronym in Portuguese for the Health Sciences Institute for Higher Education), because a good number of them were already health professionals, nurses, technicians (basic and secondary level), and we sent them to the units to provide support”.*

The government threatened the striking HCW with disciplinary proceedings on the basis of Article 95 of the Regulation of the General Statute of State Employees and Agents [46], generating some pressure on the striking group, which later contributed to end the strike.

E17 (episode interview, government representative):* "I told them: look, you're on strike, but I'll keep marking absences every day, when you reach 30 absences you will have a disciplinary process."*

E12 (doctor, strike group):* "The financial part started to weigh on us, because we weren't working, right? And clearly, the government had said that the salaries wouldn't come in and many depended a lot on this salary. So, during the negotiations, we were aware that we didn't have much time."*

At some point the government resorted to the use of police force to contain the strikers, prevent vandalism and control crowds, culminating in arrests, causing tension between the government, the strikers and some supporters (the population).

SAVANA newspaper (05–24–2013 edition): *‘’At the Beluluane Health Center, Boane district, Maputo province, patients broke down the door and removed a number of medicines. Information from Magude, also in Maputo province, indicates that the police chased away and arrested the HCW involved in the strike. The population protested against the measure, invaded the district police command and released the detainees’’  *[47]*.*

SAVANA newspaper (05–24–2013 edition): *‘’Despite several members of the executive reiterating that there is openness to dialogue, the same government used police force to prevent the doctors' meeting scheduled for this Wednesday in the Nangade garden in Maputo city center. Early in the morning, a contingent of armed police surrounded the garden and prevented the strikers from accessing the site. The strikers moved to the Costa do Sol beach where they met to outline new strategies’’  *[47]*.*

#### Tactics adopted by the strikers

One of the tactics adopted by the strikers was to establish contact with other entities that could intervene in their favor because they believed that the negotiating team representing the government did not have any decision-making power and that negotiations with them would not have the desired effect.

E04 (doctor, strike group): *"We involved other people who had influence or who could reach the leaders, like ambassadors, to go behind the scenes to unblock this, because the people we had been talking to on a day-to-day basis weren't working."*. This contributed to the government considering the existence of an external political influence on this movement.

During the negotiation process, while some were in dialogue with the government, others were marching and protesting in the streets.

E12 (doctor, strike group): *"This negotiation process was about sitting down and talking and sometimes they gave in, but then they [government negotiators] didn't comply, so we had to sit down and negotiate again, and in the meanwhile our colleagues were protesting and marching in the streets."*

There were also threats and attempts to prevent other non-striking HCW from entering the hospitals to work.

E01 (doctor, government representative): *"There was a day when they wouldn't let any workers in and threatened to remove everyone who was working there, it seemed that the aim was for patients to be left without care*."

On the other hand, they pressurized trainee students (in their final year of medical school) to join the strike by invoking the AMM Statute, which in its sixth article includes them as effective members, calling them trainee doctors [38].

E11 (doctor, government representative):* "There was some opportunism on the part of the AMM because in the Statute, final year students were called trainee doctors and, if they wanted to, they could be members of the Association."*

Due to the pressure exerted by the Faculty of Medicine during and after the first strike, the final year medical students didn't join the second strike, they were present at the internship (in the hospitals) and at some point, they had to take care of patients without proper supervision, which was opportunely denounced and criticized by the strikers.

E17 (episode interview, government representative): *‘’They used to call the students 'trainee doctors' to encourage them to join the strike, but when they saw that the students were helping to care for the sick, the discourse changed, the criticism became 'they left the sick in the hands of the trainee students', they were no longer trainee doctors, they were students".*

SAVANA newspaper (06–07–2013 edition): *''Every day there are reports of deaths in maternity wards and other wards due to lack of care or poor care by inexperienced medical students''* [14].

Jornal Notícias (06–08–2013 edition): *‘’The strike by doctors and other health professionals enters its third week today. To alleviate the absence of these professionals in caring for patients, the management of the Maputo Central Hospital (HCM) decided to use, among other resources, final year students, something that is deserving criticism from different sectors of society, with some even going so far as to consider the attitude as criminal’’  *[48]*.*

E017 (episode interview, government representative): *''We've reached the point of such desperation that we've got final year medical students doing things that only doctors can do, you see? But if we didn't do that, mortality would be higher''*.

SAVANA newspaper (edition 05–24–2013): *‘’A group of nursing students from the Maputo Institute of Health Sciences helped a 22-year-old patient bring her baby into the world at 11:30 pm this Tuesday. The labor pains began at 2pm and two hours later, the patient was already at Mavalane General Hopsital. Until 10 pm she had not been visited by any health professional. An hour and a half later, she gave birth with the help of the students” *[47].

#### Tactics adopted by mediators

The mediators assumed a pedagogical role, raising awareness and promoting dialogue between the parties.

E06 (religious leader, mediator): *"We started educating the believing doctors in our churches, sensitizing them and showing them the value and role that God has given them, and so we gradually managed to bring the medical and auxiliary staff back."*

On the other hand, the mediators recruited people (from the communities, religious groups) to provide support in the health units (in administrative and cleaning activities) as a way of relieving the workload of the available professionals.

E06 (religious leader, mediator): *"We had to bring in a lot of people, believers from the churches, to do auxiliary service in the hospitals, because the auxiliary staff also went on strike."*

Other civil society organizations—intervened in the media (through interviews and press releases) criticizing the way the strike was being managed, trying to raise awareness and bring the parties closer together, so that they would reconsider their positions to safeguard patients' rights.

*"This is no longer the time to discuss the legality or otherwise of the strike, but the seriousness of the situation leads us to seek only solutions. This requires humility on both sides in order to facilitate dialogue,"* said the civil society representative (Joint) as reported in Jornal Notícia newspaper (06–13–2013) [49].

Jornal Notícia (06–10–2013 edition): According to OrMM, the *"Guidelines for the second general strike by doctors in Mozambique contravene the profession's Code of Ethics and Deontology, which is why it called on doctors to provide minimum essential services (with ward rotations, permanent work in emergency services and the maternity ward)’’* [50].

Jornal Notícia (06–08–2023 edition): *''The executive secretary of the Forum of Non-Governmental Organizations of Gaza (FONGA) stands in solidarity with the medical profession and all staff for their demands for better salaries and working conditions, however, at this time, it is imperative to look at the precious value of human life, returning to work to make your contribution, while steps are being taken for an eventual solution to the dispute'' *[48]*.*

### Perceived impact of the strike

#### For the National Health System, services and users

With the strike, many services were limited in the public health sector, compromising even laboratory and pharmaceutical services and the follow-up of patients with chronic diseases. Many other impacts have been referred to in the previous quotations (Table 6).

**Table 6:** Perceived impact of the strike by doctors and other HCW

Impact of the strike	For the National Health System, in services and for users	• Delays in hospital care • Poor service /Low quality of services • Postponement of care • Lack of care and deaths • Fear of using public health services • Lack of medicines in health units’ pharmacies • Exhaustion of morgue capacity • Loss of trust between doctors and the government • Loss of credibility of the National Health System • Limited operation of health units
	For doctors and HCW	• Physical and psychological exhaustion of non-striking HCW • Interruption of the postgraduate program
		• Mistrust among HCW
		• Loss of empathy towards HCW
		• Expulsion, disciplinary process, transfers, compulsory retirements and arrests
	Changes achieved with the strike	• Approval and (partial) implementation of the Doctors' Statute
		• Salary increase for doctors (through subsidies)
		• Career progression and postgraduate restructuring (specialisation)

*HCW* Healthcare workers

E15 (doctor, OrMM, mediator): *‘’In this strike, the health services were practically closed. In any strike, from the outset, the services that will continue to operate must be defined, I'm talking about emergency rooms, pharmacies, laboratories, because health is not just about doctors, health is a set of activities, if the laboratory doesn't work, if the pharmacy doesn't have medicines, the presence of the doctor is almost nil, so I think these aspects were not taken care of’’.*

Savana newspaper (06–07–2013 edition): *''In the pharmacies of Maputo's central hospital, Mavalane and Jose Macamo general hospitals, as well as in some health centers on the outskirts, there is a shortage of almost all kinds of medicines, even simple painkillers like paracetamol. The situation is much more dramatic for chronic patients such as those suffering from tuberculosis, high blood pressure and HIV/AIDS. At Mavalane General Hospital, thousands of people suffering from tuberculosis come to the hospital looking for medication, but to no avail. The answer is almost the same: either the health workers aren't there or there are no medicines"* [14].

Patients had their healthcare postponed due to the strike and were forced to live with their health problems until the situation normalized.

SAVANA newspaper (05–24–2013 edition): *"A 55-year-old patient had surgery scheduled at Maputo's Central Hospital for Monday, May 20. The treatment didn't take place and he will have to endure the pain of the wounds on his face caused by skin cancer for as long as the strike lasts 'It hurts so much, it's burning, I don't know what to do, the wounds bleed and I spend sleepless nights. I can't take any medication because nothing has been prescribed. I live on painkillers to try to alleviate the pain' she complained in a conversation with our newspaper"* [47].

The strike affected the provision of health services, since it caused a greater shortage of human resources, leading to delays in care, including a lack of care for the sick and deaths.

E09 (religious leader, mediator): *"It was the people suffering, regardless of whether the doctors were right or wrong, the people were suffering, and there were images of people lying on the ground, unattended and in a state of neglect".*

*"The nurses only call people in every 20 or 30 min, and when the person comes in, they stay for a long time. I don't know what's going on, but it's not normal. We're suffering,"* said one user interviewed by *Savana newspaper (06–07–2013)* [14].

On the other hand, users complained about the impatience and arrogance of the doctors on duty, as a way of expressing their disagreement with salaries and other aspects claimed by the strikers. Some HCW adopted a kind of silent strike*.*

*"She (the patient) went to that health unit to have a gynecology consultation, but the health professional who attended her told her to go back and violently closed the office door…",* as reported by a user of the Chimoio Provincial Hospital in *Jornal Notícias* newspaper (06–19–2013) [51].

Savana newspaper (06–07–2013 edition): *‘’Although some HCW stay at their posts to guarantee minimum services, the few patients who gain courage and go to the health units told SAVANA that the staff are there just to be present and avoid absences’’* [14].

Patients had a certain fear of seeking health services, and only did so in a serious condition, which made treatment and prognosis difficult.

*"The number of patients, especially children, who arrive at the hospital at an advanced stage of illness, which makes medical treatment difficult, and ends in death, is on the rise. This shows that people, even if they are ill, prefer to stay at home",* said a non-striking doctor to Savana newspaper (06–07–2013 edition) [14].

The strike by doctors and other HCW has also affected funeral services in some hospitals.

SAVANA newspaper (05–24–2013 edition): *''Bodies left the mortuary adjacent to the HCM due to the lack of doctors to sign death certificates and allow autopsies to be carried out. By Wednesday afternoon, more than 50 bodies were waiting for autopsies to be carried out and then handed over to their relatives. SAVANA sources say that the HCM morgue is running out of capacity to hold more bodies’’  *[47]*.*

The strike created a climate of distrust between HCW and the government, and added to this, the National Health System was discredited by the people.

*E01* (doctor, government representative)*: "Patients lost faith in the health services, there was a great discrediting of the Health System, and they were afraid to even visit the health units."*

#### For healthcare workers

As part of the strike, postgraduate programs (specialization) were interrupted due to a shortage of human, material and financial resources [52]. As a result of the work stoppage, disciplinary proceedings were initiated because of unauthorized absenteeism, which resulted in fines, demotions, dismissals, transfers, arrests and compulsory retirement [53].

*E02* (doctor, government representative)*: "All (of the strikers) were instructed in disciplinary proceedings, and according to the outcome of each case, decisions were taken".*

*E04 (doctor, strike group): "There were many threats and reprisals, and the president of the association was arrested, and some colleagues were dismissed and lost their management positions".*

On June 5, 2013, the MISAU decreed the suspension of post-graduate training in the National Health System due to a shortage of human, material and financial resources, and doctors undergoing post-graduate training would be reallocated to health units throughout the country [52].

This decision represented a setback in the lives of the strikers, most of whom were in postgraduate studies, which provoked revolt and created a moment of tension between MISAU, OrMM and AMM. The colleges of obstetricians and gynecologists, and dermatologists issued statements expressing their displeasure with the decision and demanding the reinstatement of the program (SAVANA edition 06–14–2013) [54].

According to the AMM representative (SAVANA edition 06–21–2013): *‘’some doctors were disappointed with the decision, some thought they would return, but many of those who wanted to return to work before the end of the strike were not received, because the specialty colleges did not agree with the MISAU's action to stop postgraduate studies. This measure taken by the MISAU is illegal, because the issue of postgraduate studies should not be dealt with unilaterally by the Ministry, it needs a joint commission with the OrMM’’  *[55]*.*

The oldest doctors and founders of the National Health System (85 specialists, including former ministers of health, previously advisors to the minister of health) wrote and made public a letter addressed to the president of the republic expressing support for the striking doctors and their indignation and revolt regarding the working and living conditions of HCW, especially young doctors and postgraduates. They also condemned the acts of coercion, intimidation, dismissal and repression exercised against striking HCW and the use of students, rescuers and volunteers to meet the demand for services, classifying this act as an illegal practice of medicine (Jornal Notícias, edition 06 −08–2013) [48].

On the other hand, civil society felt that HCW had lost their professionalism and patriotism, which contributed to part of the community losing empathy and trust in them.

E06 (religious leader, mediator): *"A social situation of mistrust towards health professionals has been created, there have been serious consequences in relation to patient care that have greatly undermined the ethical recognition of HCW".*

For the non-striking doctors and HCW, there was an overload of work that resulted in exhaustion and demotivation.

One of the students from the Maputo Institute of Health Sciences assigned to provide support at Mavalane General Hospital told SAVANA that people are working overtime and some of her colleagues are already complaining of tiredness*: "My roommate came in at 10am on Monday and didn't leave until 12 pm on Tuesday. She's going to rest all afternoon and evening, because she has to return at 7am tomorrow (Wednesday). I have many colleagues in this situation. It's very complicated. I think that even people who have been doing these routines for years wouldn't be able to deal with it" (SAVANA newspaper, 05–24–2013 edition) *[47]*.*

Savana newspaper (06–07–2013 edition): *''Doctors who guarantee care in hospitals are exhausted and are threatening with a public letter denouncing the chaos in health units''* [14].

#### Changes after the strike

After the strike, the Doctors' Statute was approved and implemented with the corresponding allowances, which in a way led to an improvement in doctors' salaries. Career issues and the right to housing were also supposedly safeguarded by the Statute, once approved.

E07 (doctor, strike group): *“Looking at it with a cool head, we had a different salary increase, because that year the salary increase for all public services was 7.0 or 7.5% and we had a 15% increase, and the following year the same. We had our Statute approved in 2013, some aspects of the Statute began to be implemented in 2016, so three years later, others had to go to the Administrative Court, so they were only implemented in 2021”.*

## Discussion

### Contextualisation

The strike by public sector HCW in Mozambique in 2013 was an unprecedented protest movement, which initially involved only doctors (10-day strike in January), but took on a greater dimension as agreements were not reached with government, culminating in the involvement of other HCW groups in month-long strike in May and June, thus becoming a generalized strike in the public health sector.

Within the framework of the strike, agreements were reached, but many of these pacts are still subject for debate and disagreement factors for perpetuating dissatisfaction and maintaining mood of revolt. These factors contributed for a more recent strikes, especially because of deficient implementation of the Statute of Doctors in Public Administration. Since the end of 2022, the country has been facing successive strikes (by doctors and other HCW) in the public health sector including dissatisfaction over the salary reforms implemented by the government in 2022, which clashed with the terms agreed in the Statute of Doctors in Public Administration following the 2013 strike, which in turn, according to the doctors, was never fully implemented [56]. This close look at the 2013 strike in terms of its causes, contours and consequences from the perspective of the main actors involved, may help to shed light on how to improve the means to address the issues that persist and help to prevent further strikes in the future.

This strike in Mozambique, follows a previous undocumented strike by doctors in 1989. It took place at a time when there were strike movements by HCW in neighboring countries (such as Tanzania and Madagascar in 2012) and all over Africa (such as Niger, the Democratic Republic of Congo, Liberia, Kenya, and Burkina Faso in 2013) [4, 57], and there may have been some cross-country influence among all these movements in different SSA countries. In Mozambique, as in other countries [4], the strike movement was led by a trade union or medical association and assumed contours that were not always supported by the Medical Councils.

### Causes of the strike

The main factors motivating the strike of doctors and other HCW in Mozambique were related with salaries and working conditions. The lack of communication and trust between the parties, and the failure of health authorities as employers to fulfil previously signed agreements, precipitated the subsequent strikes.

These results corroborate the findings of studies carried out in other countries [1, 5, 8, 58–63], and in Kenya in particular, that reflects government’s failure to fulfil agreements reached with the HCW (doctors and nurses) to improve remuneration (salaries and allowances), enhance human resources capacity and equipment availability in health facilities; these triggered a sequence of strikes by doctors, nurses and clinical officers during 2017 [1, 59, 60, 62].

Salary demands sometimes arise by comparison with the private sector or other public sectors but with different privileges. The salary levels of these other privileged civil servants were, according to the present study, taken as a reference by strikers-leadership to advocate for better remuneration for HCW, as shown also in a study carried out in Malawi (in 2001), where doctors used the salaries of the judiciary as a reference, while in Niger (2017), junior doctors used the salaries of specialists doctors to demand better salaries [4, 6]. Hence salary reforms in the public sector needs to consider the overall public servant salary structure and minimize unreasonable disparities.

### Approaches adopted during the strike, its contours and impact

The government of Mozambique, represented by MISAU, as a way of stopping the strike, created a mixed team to negotiate with AMM. Initially, a consensus was reached regarding the need to increase doctors' salaries, but there was disagreement regarding how this could be done. The government was worried that its decisions would trigger a generalised strike (involving the police, teachers, and other professional classes), so they opted for a discreet salary increase mechanism, by means of allowances. These were specified in the Statute of Doctors in Public Administration, which would be regularised and implemented in stages. However, factors such as lack of communication, political interference and questionable criteria for managing public funds were pointed out by the string workers as contributing to a climate of mistrust and intolerance between the parties, which made the negotiation process challenging. On the one hand, the government was asking for more time to regulate and implement the Doctors' Statute (including allowances), while on the other the doctors were intolerant and inflexible with the proposed deadlines that were relatively long, hence, the strike continued, and access to health services remained compromised.

The National Health System was facing a critical shortage of human resources, with a ratio of one doctor per 17,235 inhabitants, far from that recommended by the World Health Organization [16, 64], and with the strike, the situation became worse, causing overload and exhaustion of available HCW, leading to critical challenges in health services, compromising the availability and the quality of the health care provided. However, in order to mitigate the effects of the strike, the government opted to call for an alternative workforce (such as military doctors, health managers, medical students and students from other health courses) and ask for help from the Mozambican Red Cross, communities and religious groups to provide support in health units with shortage of HCW. This approach has been adopted by other governments in similar situations, as shown by studies carried out in Kenya and Malawi [1, 61–63], which brings some relief to the workload of non-striking HCW.

The inclusion of religious leaders as mediators in the dialogue between the parties was a tactic of the government, which sought, through them, to sensitise the striking doctors who, as well as being HCW, were also believers, calling on them to think about the patients suffering in hospital queues and to look at the purpose of their profession. This tactic seeks to exploit the influence that religion has on well-being and social behaviour, promoting a set of self-regulatory behaviours, as explained by McCullough and Willoughby [65], thus taking advantage of the fact that the strikers profess these religions and would eventually be more sensitive to appeals from religious leaders.

However, religious leaders were also trying to recover the apparent loss of sensitivity that doctors had suffered in the eyes of civil society. The trust and empathy that society had for doctors and other HCW was shaken, and the National Health System, in turn, was discredited, as was also reported in studies carried out in Nigeria [8, 9] and Kenya [1].

The striking group included were doctors in post-graduate training (medical specialisation) who were affected as their programmes were interrupted, and as an aggravating factor, disciplinary proceedings were launched, resulting in fines, demotions, dismissals, transfers, arrests and compulsory retirements, corroborating the results of studies carried out in Nigeria and Kenya [5, 61].

Therefore, although there were changes after the strike, with the regulation and implementation of the Statute of Doctors in Public Administration (with risk and seniority allowances, as well as the right to housing included), all parties involved lost with the strike, especially the patient. It should be taken into account that a large part of the Mozambican population is poor and reside in rural areas and cannot afford to access private healthcare services, mostly prevalent in urban areas (in 2009 the incidence of poverty was 54.7 per cent). The public sector is the only option for accessing healthcare for many [66, 67]. Therefore, when the public health sector goes on strike, a large part of the Mozambican population is prevented from accessing formal healthcare. This is also well described from other strike movements in Africa [68].

### Reflections around the strike in the healthcare sector and recommendations

The legitimacy of the strike has always been a controversial issue, although it is allowed by law as a fundamental right of workers, according to article 87 (1) of the Constitution of the Republic of Mozambique of 2004. However, the provisions of article 87( 2) limits the exercise of the right of strike by essential services and activities, by workers from the unavoidable needs of society and national security [69]. The same law, in its articles 40 and 89 guarantees citizens the right to life and physical and moral integrity, free from torture or cruel and inhuman treatment, and the right to medical and health care, respectively. Not forgetting the Code of Ethics of the Medical Council [70], Article 6 (1) which states that "doctors must exercise their profession with the utmost respect for the right to health of patients and communities", and Article 10 which states that "in the event of a doctors' strike, and whatever the circumstances, doctors must ensure the continuity of therapeutic care for their patients, as well as assistance to urgent and seriously ill patients”.

However, during the second strike of doctors and other HCW in 2013 in Mozambique, according to the strike guidelines released by AMM, minimum services were the responsibility of foreign and military doctors and of doctors with management positions who, due to the inherent nature of their functions and contractual aspects, could not join the strike. Therefore, the exercise of the strike is a constitutionally guaranteed right, but it is conditional on the provision of minimum services, so the strike guidelines clash with this last precept, since the strikers delegated the minimum services to others [13, 37].

There are several reported strategies used around the world for similar strikes to minimize harm to patients and protect their rights. The "urgent and emergency care" industrial action model adopted by the British Medical Association has been an example to follow as it ensures that patients receive care in a real emergency situation [71]. This model advises that doctors, although on strike, should be at their usual place of work providing care in emergency or urgent cases, and since it is not always clear how serious the case is, in case of doubt, it is recommended that care be provided. Therefore, the strike is limited to non-urgent cases only. This model has also been implemented in Tanzania, Australia and Israel, where an alternative "fee for service" system was also created to ensure that patients had healthcare options [71]. Another strategy that has been adopted by governments in similar situations is to seek greater cooperation with the private sector, including relieving fees, providing supplies and referring cases for hospitalization, thus relieving the burden on public health units and preserving the well-being of patients [1, 62, 68, 72].

No matter how just the struggle or the demand for our rights may be, "life" must be considered as the most precious asset. The motto of the MISAU says it all: "Our greatest value is life". There is a need for deep reflection at all levels on how this class can claim its rights when its services and activities are essential to the well-being of the citizens.

On the other hand, it is understood that each profession has its specificities and requirements. Doctors are required to have good character, integrity, compassion, altruism, continuous improvement, excellence, teamwork and selflessness for the benefit of humanity, with patient care being above all else, including their own wants [73]. A lot is demanded from doctors because they deal with human life. It is understandable that these HCW deserve better remuneration, so that they feel motivated. The motivation of HCW is directly linked to the quality of the services they provide. Therefore, motivating HCW means ensuring better care for the population. Studies have shown that remuneration is a determining factor for the satisfaction of HCW, and in turn for the quality of services [74, 75]. The adoption of sustainable, fair, and transparent remuneration models in the public service is crucial to overcoming the ghosts of strikes and retaining staff.

Disputes or conflicts in the workplace are inevitable and are not always destructive. They can be constructive depending on how they are managed, which depends on the organizational conflict culture adopted. There are three types of conflict culture: I) collaborative conflict cultures, in which collective constructive dialogue, negotiation and joint problem solving prevail; II) dominant conflict cultures, in which the members of the organization collectively seek competition and victory, and try to outwit each other, and III) avoidant conflict cultures, in which the members of the organization collectively oppress and stay away from the problem [76].

The intimidating or punitive actions taken by the government, together with the lack of fluidity in the dialog between the parties, caused the conflict to reach undesirable proportions, with accusations, mistrust, distancing between the parties, and a climate of tension established. This may not be the most appropriate way to resolve this type of conflict, as it could have the opposite effect to that desired [3], generating judicial actions, disputes, mistrust, distance and resentment between the parties, and historical marks that are difficult to overcome. As seen, years have passed since this strike occurred, but it is still the result of debate, controversy and disagreements, being identified by many as the genesis of strikes in the National Health System in Mozambique [77].

It was evident that the strike has damaged the relationship between all stakeholders (managers, striking HCW, non-striking HCW, patients, and civil society), creating distrust in the health sector. Distrust undermines interpersonal relationships and teamwork and is a strong catalyst for more labour conflicts and repulsion towards public health services on the part of the community [78]. It is essential to adopt a culture of collaborative conflict within the workplace and permanent dialogue between the parties is the key, not only to resolving the strike, but also to maintaining a healthy, productive and innovative working environment.

It is important to restore trust and adopt a mechanism to preserve it. A more participatory leadership model, involving different stakeholders in decision-making, such as governments, universities, civil society, the private sector, the media and voluntary groups (including religious organisations and patient groups), in addition to the communities themselves, ensures that groups share concerns and responsibilities, which can generate better understanding and sensitivity to difficulties, and manage expectations, directly impacting on group satisfaction, motivation and behaviour [79, 80].

There is also a need to strengthen leadership in human resource management and labour conflicts in health, so it would be opportune to introduce a master's level course in human resource management and leadership for health, aimed primarily at public health service managers. Studies that identify the countries with the least occurrence of strikes in the health sector and analyse the factors that contribute to this fact and the management tactics adopted in these countries would be of vital importance.

With regard to working conditions, it is important to note that health care is the result of a joint effort and shared responsibility between HCW, the government and society, and that everyone's commitment to its provision is essential. The government and society have a responsibility to provide the necessary means for HCW to operationalize health services [2].

Therefore, future intervention studies aimed at promoting humanisation in the health sector are fundamental, as are studies evaluating and monitoring the satisfaction and motivation of HCW in the workplace and mechanisms to ensure an ongoing constructive dialogue with HCW. Any information that allows the government to act in advance to address issues that may result in HCW’s strike situations is useful.

### Study limitations

However, this study had some limitations: the phenomenon under study (strike) took place 10 years ago (2013), so there is a risk of memory bias. However, the inclusion of document analysis and member checking may have minimized the likelihood of its occurrence, thus giving the data greater reliability. On the other hand, the non-inclusion of private health sector stakeholders limits the understanding of the implications of the strike, since according to Yoong et al. [81], private sector participation is positively associated with better functioning of the health system in terms of access and equity and may provide overflow flow care for patients not received in the public sector. Therefore, future studies that address the HCW' strike from the perspective of private sector managers would be important, as well as studies with a quantitative approach aiming to analyze the impact of the strike considering health indicators such as mortality, number of patients treated, hospitalizations and surgeries performed.

## Conclusions

This study represents an opportunity to reflect on the causes and consequences of strikes in the health sector, as well as on the ways to strike in a sector that is so crucial to the well-being of society. It also represents a starting point for drawing up effective strategies and policies for managing and preventing labor conflicts in the health sector. The strike by doctors and other HCW in Mozambique in 2013 was the first major strike movement in the history of the public health sector in Mozambique, due to its duration and impact. The main causes of this strike were issues related to salaries and working conditions. The end of the strike was not entirely peaceful and the contours were marked by a lot of tension between the parties. Disagreements regarding wages and working conditions were not completely resolved maintaining grievances even after resuming of work by doctors and other HCW. All approaches apparently were for short term gain, but weak on reflection to deepen understandings necessary to ground sustainable strategies for the future.

## Supplementary Information

Supplementary Material 1. Interview Guide

Supplementary Material 2. Supplementary Table 1

Supplementary Material 3.

## Data Availability

The datasets used and analyzed during the current study are not publicly available due identifiable information but are available from the corresponding authors upon request.

## Abbreviations

HCW: Healthcare Workers
MISAU: Ministry of Health
AMM: Mozambique Medical Association
OrMM: Mozambican Doctors' Council
HCM: Maputo Central Hospital
FONGA: Forum of Non-Governmental Organizations of Gaza
MoU: Memorandum of Understanding
ANEMO: National Association of Nurses of Mozambique
ATLAC: Association of Laboratory Technicians and Clinical Analysis
PSU: United Health Professionals
OTM-CS: Mozambican Workers’ OrganizationCentral Trade Union
NGO: Non: Governmental Organizations
