# Supplementary material for: A look back at the strike by Mozambican doctors in 2013: what can we learn?
Source: BMC Health Serv Res. 2024 Nov 29;24:1510. doi: 10.1186/s12913-024-11998-7 (PMC11607957; doi:10.1186/s12913-024-11998-7)
Supplement: Supplementary file 1 — Supplementary Material 1. Interview Guide [file 12913_2024_11998_MOESM1_ESM.docx]

**Interview Guide**

**I. Socio-demographic data**

1.1 Age_______

1.2 Gender ______

1.3 Group belonging during the strike:_____________

a) Government representative _______ b) Strike group _____ c) Mediator _____

1.4 Professional category:_________________

**2. General aspects**

2.1 When you think about the 2013 strike by doctors and other healthcare workers in Mozambique, what comes to mind?

2.2 What were the most delicate moments you experienced during the strike? What impacted you the most?

2.3 Which actors would you highlight as key in the management of the strike movement of 2013?

2.4 What lessons can be learnt from that strike movement?

**3. Causes**

3.1 What do you think were the real motivations (causes) of that strike movement in 2013?

3.2 Is there any political or social aspect, in your opinion, that contributed to that strike taking place?

3.3 What is your opinion of the demands book, were the points presented justified at the time?

3.4 Which aspects contributed to the outbreak of the strike movement?

**4. Strategies**

4.1 What strategies or interventions were adopted by the parties to prevent the strike from taking place?

4.2 What do you think should have been done and wasn't done (by the parties) to prevent the strike from starting?

4.3 What interventions were made to mitigate the effect of the strike? What implications did they have?

4.4 What interventions were made to stop the strike? What implications did they have?

4.5 What strategies did the strikers adopt to achieve their goals?

4.6 What really contributed to the end of the strike movement?

**5. Consequences**

5.1 What were the consequences of that strike movement for patients, the health sector and the government in general?

5.2 What consequences did the strike have for doctors and other healthcare workers (strikers and non-strikers)?

5.3 Has the government met all the points contained in the list of demands?

5.4 What is your assessment of the living and working conditions of doctors and other healthcare workers before and after the strike?

5.5 How was this strike reported in the media and what was public opinion regarding the movement?
