# Supplementary material for: A look back at the strike by Mozambican doctors in 2013: what can we learn?
Source: BMC Health Serv Res. 2024 Nov 29;24:1510. doi: 10.1186/s12913-024-11998-7 (PMC11607957; doi:10.1186/s12913-024-11998-7)
Supplement: Supplementary file 2 — Supplementary Material 2. Supplementary Table 1 [file 12913_2024_11998_MOESM2_ESM.docx]

Table S1: List of documents included in the document analysis.

| **Ord** | **List of documents included in the analysis** | **N** |
| --- | --- | --- |
| 1 | Jornal Notícia Newspaper (diary) | 15 |
| 2 | SAVANA Newspaper (weekly) | 08 |
| 3 | Health Human Resources Annual Report 2013 | 1 |
| 4 | Health Human Resources Annual Report 2021 | 1 |
| 5 | Pre-Notice of General Doctors Strike (2012) | 1 |
| 6 | Pre-Notice of General Strike for Doctors and healthcare workers (2013) | 1 |
| 7 | Doctors' claim book (2012) | 1 |
| 8 | Doctors' claim book (2013) | 1 |
| 9 | United Health Professionals Claim Book (2013) | 1 |
| 10 | General report on the paralysation of activities in the National Health Service by doctors and other healthcare workers (2013) | 1 |
| 11 | Memorandum of Understanding between the Ministry of Health and the Medical Association of Mozambique (2013) | 1 |
| 12 | Regulation of the Statute of the Doctor in the Public Administration (2014) | 1 |
| 13 | Statute of the Doctor in the Public Administration (2013) | 1 |
| 14 | Guidelines for the General Healthcare Workers' Strike in Mozambique (2013) | 1 |
| 15 | Guidelines for the 2nd General Doctors' Strike in Mozambique (2013) | 1 |
| 16 | Regulation of the Medical Association of Mozambique (2015) | 1 |
| 17 | Orders issued by the Minister of Health between | 06 |
| 18 | Book of minutes of meetings between MISAU and AMM | 12 |
| 19 | Book of minutes of the meetings of the Executive Board of the Ministry of Health | 8 |
| 20 | Report of disciplinary proceedings raised within the scope of the work stoppage (2013) | 1 |
| 21 | Constitution of the Republic of Mozambique of 2004 | 1 |
| 22 | Regulation of the General Statute of State Employees and Agents (2009) | 1 |
| 23 | Statute of the Mozambican Doctors Council and Code of Ethics (2011) | 1 |
| 24 | Pedagogical Regulations of the Eduardo Mondlane University (2010) | 1 |
|  | Total | 68 |
