# Supplementary material for: A look back at the strike by Mozambican doctors in 2013: what can we learn?
Source: BMC Health Serv Res. 2024 Nov 29;24:1510. doi: 10.1186/s12913-024-11998-7 (PMC11607957; doi:10.1186/s12913-024-11998-7)
Supplement: Supplementary file 3 — Supplementary Material 3. [file 12913_2024_11998_MOESM3_ESM.docx]

**Reflexivity of the research team**

Recognising the range of internal (personal and interpersonal) and external (political or social) influences on the research, given the subject of the research, the research team adopted a reflexive and collaborative approach to identify and mitigate biases, so that the research was conducted in a balanced, transparent and ethical manner.

**1. Team composition**

The team began the project by holding group discussions about their experiences and positions in relation to the health professionals' strike. All the members of the research are connected to health in some way, with four of them being doctors (A.L.J.M., P.F., A.J.R.C. and M.S.), a sociologist with a mastery of qualitative research methods in health and health promotion and education (I.C.) and an optometrist and pedagogue (D.B.S.).

Among the members, two (A.L.J.M. and M.S.) experienced the study event as members of the government and managers, so it is natural for them to have a dissenting tendency towards the strike movement, while the other members, being doctors, health professionals and involved in the training of health professionals, have a natural tendency to sympathise with the movement. Therefore, there was a need to maintain a balance of forces in order to guarantee impartial results. From the group's initial discussions, the need to look for strategies to neutralise tendencies and balance perspectives was emphasised.

**2. Diversification of perspectives within the team and control strategies.**

Researchers A.L.J.M., P.F., I.C. and M.S. designed and revised the methodology and interview guide, which was shared and reviewed by a panel of experts to ensure that the questions are not biased and inclined to favour one side. In this way, they revised the data collection instrument to ensure that all parties involved - strikers, government and mediators - are approached in a balanced way.

On the other hand, a researcher from outside the study with some experience in qualitative studies was trained to carry out the interviews and ensure the utmost neutrality.

The data had to be analysed by three researchers (A.L.J.M., M.S. and D.B.S.), one of whom had some distance from the strike (D.B.S.) to ensure a certain balance in the analysis. Initially, the data was analysed individually to avoid mutual influence between the researchers. Subsequently, they had to review the transcripts together and discuss how each one interpreted and coded the participants' speeches.

During the compilation of the results, analysis and discussion, for the first author (ALJM), as main author, and who at the time of the facts was Minister of Health and had the responsibility of defending the government's position that saw the doctors' demands as unjustifiable given the country's condition, it was difficult to adopt a more neutral position due to his involvement as a member of the government. Therefore, the first analyses he led tended to impute the consequences of the strike to the striking doctors, but with the intervention of the other members of the team (P.F., I.C., A.J.R.C. and D.B.S.) a more balanced approach was obtained and the sharing of responsibilities brought into the discussion.

The team believes that the analysis carried out in this study could be used to justify policies for or against the strike. Therefore, the study should be conducted in a responsible, fair and balanced way, so as not to favour one side over another.
